# A rare case of secondary small bowel volvulus laparoscopically repositioned: literature review and classification

**DOI:** 10.1186/s40792-018-0470-z

**Published:** 2018-06-27

**Authors:** Koichi Inukai, Hidehiko Kitagami, Shuhei Uehara, Hirotaka Miyai, Nobuhiro Takashima, Minoru Yamamoto, Kenji Kobayashi, Moritsugu Tanaka, Tetsushi Hayakawa

**Affiliations:** 1 Department of Surgery, Kariya Toyota General Hospital, 5-15 Sumiyoshi-cho, Kariya, Aichi 448-8505 Japan; 2 Department of Laparoscopic Hernia Center, Kariya Toyota General Hospital, 5-15 Sumiyoshi-cho, Kariya, Aichi 448-8505 Japan

**Keywords:** Small bowel volvulus, Laparoscopy, Free jejunum flap

## Abstract

**Background:**

Secondary small bowel volvulus is a rare condition caused by adhesions after laparotomy or tumors. There are no clear guidelines for indication of laparoscopic surgery.

**Case presentation:**

A 69-year-old male visited our hospital complaining of epigastric pain. He had a history of hypopharyngeal carcinoma treated via pharyngolaryngoesophagectomy with restoration of esophageal continuity by harvesting a free jejunal autograft 6 years ago.

Enhanced computed tomography revealed the whirl sign. An emergency laparoscopic operation was performed following a diagnosis of small bowel volvulus. This revealed rotation of the whole small bowel, involving the superior mesenteric artery as the center, and originating at the adhesion of the proximal and distal small bowel. Laparoscopic manipulation of volvulus and lysis of the adhesion were performed. The patient’s postoperative course was uneventful, and he was discharged on hospital day 5.

**Conclusions:**

Laparoscopic surgery may be useful for treating small bowel volvulus; however, the patient’s treatment indications should be judged carefully.

## Background

Secondary small bowel volvulus is a rare condition reported to be caused by adhesions after laparotomy or tumors. As a complication after harvesting of free jejunum flap, secondary small bowel volvulus is especially rare [1]. There are no clear guidelines for indication of laparoscopic surgery. We experienced a case of secondary small bowel volvulus, which was successfully treated laparoscopically. Herein, we present this case, particularly based on literature discussion, and investigate the indications and points to consider during laparoscopic treatment.

## Case presentation

A 69-year-old man developed a sudden epigastric pain. He was presented at this hospital as an emergency outpatient. Six years earlier, he underwent laryngoesophagopharyngectomy, bilateral lymph node dissection for hypopharyngeal cancer, and esophageal reconstruction with a free jejunum flap. On physical examination, the abdomen was flat and soft with tenderness in the epigastric region, but no sign of peritoneal irritation. Blood biochemistry findings revealed elevated values: creatinine, 1.16 mg/dl; lactate dehydrogenase, 364 U/l; and creatine phosphokinase, 622 U/l.

**Fig. 1:**
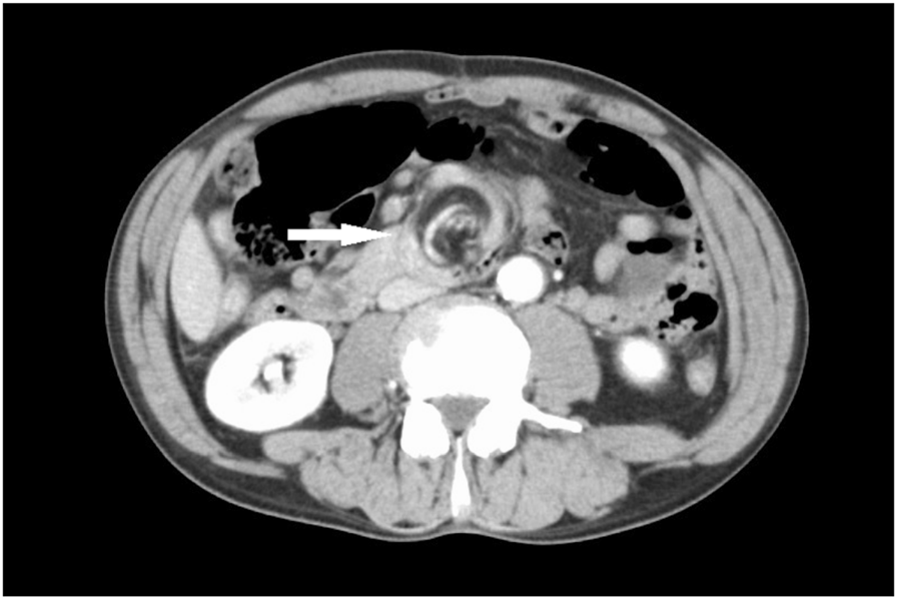
Enhanced abdominal CT shows the whirl sign (arrow) involving the superior mesenteric artery as the center

Abdominal contrast computed tomography (CT) revealed twisted mesentery with the small intestine around the point of torsion (whirl sign) and the superior mesenteric artery as the axis. Contrast enhancement was weakened in the same area of the small bowel (Fig. 1). Given this information, we suspected small bowel volvulus and performed emergency surgery on the same day.

### Surgical findings

**Fig. 2:**
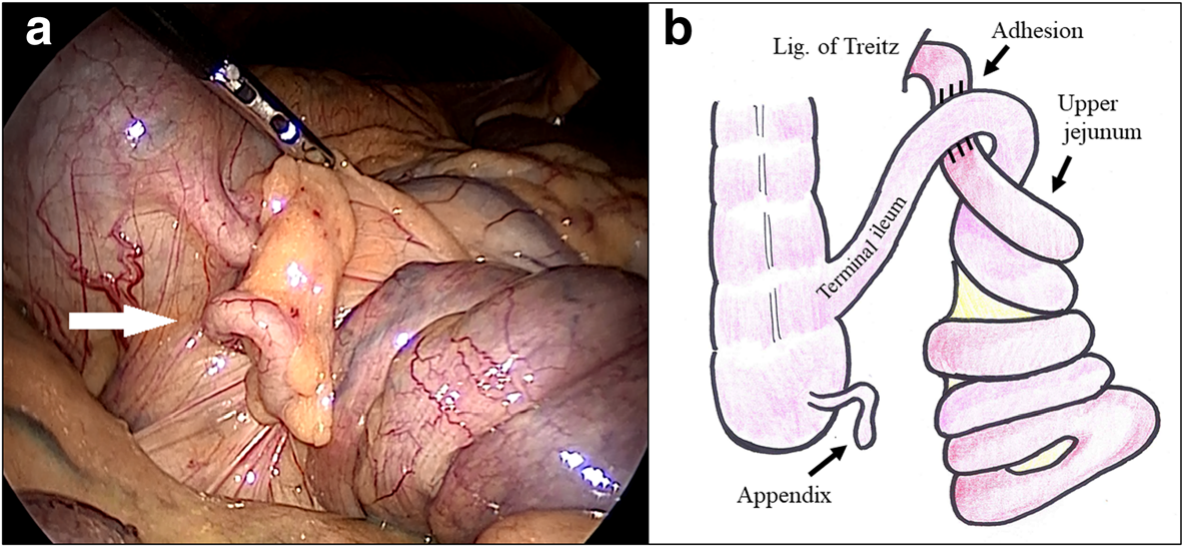
Intraoperative findings. **a** Some congestion is observed in the volvulus small bowel. The white arrow indicates the appendix. **b** Intraoperative schema showing a small bowel volvulus starting from the adhesion of the jejunum and terminal ileum

A 5-mm camera port was placed in the umbilicus and 5-mm ports in the lower and right lower abdomen. During laparoscopic examination, the upper jejunum adhered to the small bowel close to the terminal ileum with overlapping of the small bowel. The entire part from the upper jejunum to the terminal ileum was twisted clockwise with the superior mesenteric artery and vein as the axes and the adhesion site as the starting point. There were areas of poor color enhancement throughout the twisted section of the small bowel (Fig. 2). We laparoscopically separated the adhesion between different sections of the intestinal tract and traced the bowel from the small bowel in the region of the ligament of Treitz toward the anus to confirm the absence of adhesions or torsion up to the terminal ileum. The color of the small bowel improved; hence, the surgery was completed without resecting any part of the intestine.

Postoperatively, the patient made good postoperative recovery, resumed oral intake on day 2, and was discharged on day 5 after surgery. No recurrence has been reported 1 year postoperatively.

## Discussion

Small bowel volvulus is often congenital. It is caused by malrotation of the intestine or incomplete mesenteric fixing or a primary condition without an underlying disease or anatomical anomaly. Secondary cases caused by acquired factors, such as postoperative adhesions or tumors, only account for 19.5% [2]. Abdominal contrast CT is effective for diagnosis, with the intestine wrapping around the mesenteric blood vessel in a spiral, forming a characteristic whirl sign [3, 4].

We searched for secondary small bowel volvulus requiring surgery using the Japan Medical Abstracts Society (Ichushi) between 1990 and January 2018 using “secondary” and “small bowel volvulus” as keywords, and we found 29 reports (Table 1). Of these cases, 12 cases of small bowel volvulus were caused by postoperative adhesions, similar to the present case. All 12 patients underwent laparotomy prior to developing the condition, with gastrectomy as the most common procedure. Other than our case, there were no reports of the condition developing after harvesting free jejunum flap. Of 10 cases, nine (excluding two unknown cases) presented with the whirl sign on CT, making preoperative diagnosis possible.

**Table 1:** Review of secondary small bowel volvulus cases reported in Japan

Case	Author	Year	Age	Sex	Histroy of abdominal surgery	Cause of volvulus	Surgical procedure	Whirl sign	Classification of volvulus	Bowel necrosis	Complications
1	Ii	1993	60	M	Appendectomy	Mesenteric lipoma	Laparotomy	+	A	–	None
2	Takeuchi	1993	66	F	Gastrectomy	Adhesion after laparotomy	Laparotomy	+	B	–	None
3	Sakamoto	1994	51	M	Distal gastrectomy	Adhesion after laparotomy	Laparotomy	NA	C	+	Intestinal hurry
4	Kakihara	1997	58	F	None	Ileal lipoma	Laparotomy	NA	A	–	NA
5	Tani	1998	34	F	None	Ileal lipoma	Laparotomy	+	A	–	None
6	Taka	1999	83	F	Gastrectomy	Incarcerated femorall hernia	Laparotomy	+	B	–	None
7	Kawasaki	1999	83	F	Appendectomy	Adhesion after laparotomy	Laparotomy	+	B	–	None
8	Nakamura	2002	53	F	None	Small intestinal GIST	Laparotomy	+	A	–	NA
9	Hisada	2002	67	M	None	Adhesion after laparotomy	Laparotomy	+	B	–	None
10	Yamamoto	2002	84	F	Distal gastrectomy, cholecystectomy, hysterectomy	Adhesion after laparotomy	Laparotomy	+	B	–	NA
11	Yoshida	2003	26	M	None	Traumatic hematoma	Laparotomy	+	A	–	NA
12	Uchiyama	2003	80	M	Distal gastrectomy	Adhesion after laparotomy	Laparotomy	+	NA	–	None
13	Uchiyama	2004	87	M	Cholecystectomy	Adhesion after laparotomy	Laparotomy	+	NA	+	Sepsis
14	Minato	2004	83	F	None	Phalomesenteric duct remnant	Laparotomy	–	B	–	None
21	Yada	2004	47	M	None	Small intestinal GIST	Laparotomy	NA	A	–	None
15	Okada	2005	67	F	Cesarean section	Mesenteric cyst	Laparotomy	+	A	–	None
16	Kato	2005	77	F	Cholecystectomy	Jejunal diverticulum	Laparotomy	+	B	–	None
17	Meguro	2006	79	F	Hysterectomy	Small intestinal GIST	Laparotomy	+	A	–	NA
18	Waranabe	2006	81	M	None	Jejunal GIST	Laparotomy	+	A	–	None
19	Uzuki	2007	81	M	None	Jejunal GIST	Laparotomy	+	A	–	None
20	Nakashima	2008	64	M	None	Ileal GIST	Laparotomy	NA	A	–	None
22	Harada	2010	86	M	None	Incarcerated inguinal hernia	Laparotomy	NA	B	–	None
23	Ito	2011	26	M	Bladder augmentation	Adhesion after laparotomy	Laparotomy	–	NA	+	None
24	Tanaka	2012	35	M	Total gastrectomy	Adhesion after laparotomy	Laparotomy	+	B	–	None
25	Tanaka	2012	72	M	Total gastrectomy	Adhesion after laparotomy	Laparotomy	+	B	–	Delirium, Wound dehiscence
26	Tanaka	2012	66	M	Total gastrectomy	Adhesion after laparotomy	Laparotomy	NA	B	–	None
27	Kuroda	2013	81	M	None	Idiopathic intussusception	Laparotomy	NA	A	+	None
28	Sakai	2016	78	F	Colectomy	Adhesion after laparotomy	Laparotomy	+	B	–	Surgical site infection
29	Niwa	2017	51	M	None	Intussusception by lipoma	Laparotomy	+	A	–	None
30	Our case	2018	69	M	Harvesting free jejunal autograft	Adhesion after laparotomy	Laparoscopy	+	C	–	None

*NA* not available

**Fig. 3:**
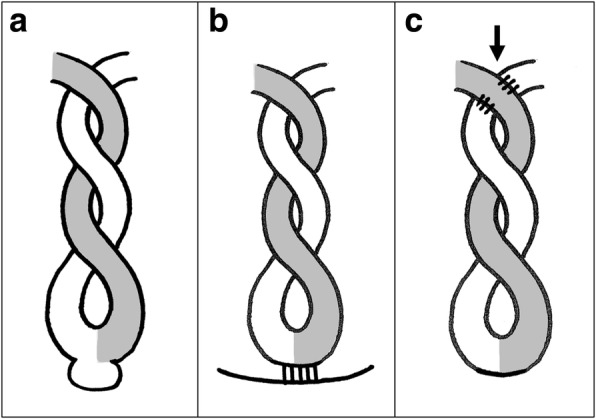
The classification of volvulus. **a** The proximal and distal bowel are twisted like a torsion pendulum by an abnormality such as a tumor. **b** The proximal and distal bowel are rotated on an axis formed by a fixed point on the abdominal wall. **c** Volvulus occurs due to the adhesion (arrow: torsion origin) of the proximal and distal bowel or the adhesion of the bowel and mesocolon

When we examined the cause of the torsion in the 29 cases, many cases (*n* = 13) had organic anomalies such as small bowel tumors or small bowel intussusception prior to the torsion, and these cases were classified as type A (Fig. 3a). The second most common presentation (*n* = 12) was partial adhesion of the small bowel to the abdominal wall or a strangulated hernia, with the same site forming a fixed site, and the bowel twisting around that site as the axis, and these cases were classified as type B (Fig. 3b). In the present case, adhesion between two different parts of the small bowel formed the origin of the torsion, with the distal and proximal sides of the small intestine twisting upon itself. A similar form of volvulus was reported by Sakamoto et al. There was only one case where adhesion between the upper jejunum and the transverse mesocolon formed the origin of the torsion [5]. In this paper, this special form of torsion was classified as type C (Fig. 3c). In type C, the torsion origin is formed due to the adhesion of the proximal and distal bowel or the adhesion between the bowel and the mesocolon, with peristaltic torsion forming in the intestine between the two points. This is considered a rare form of volvulus.

Emergency surgery was required to treat this case. The ileus is removed with reduction of the torsion, and if intestinal necrosis is suspected, small bowel resection is required. Of the 29 cases, only four had intestinal necrosis. Recently, laparoscopic examination and laparoscopic surgery have been proactively performed for small bowel obstruction, and reports indicate that the outcomes are better than laparotomies in terms of improved postoperative intestinal motility and duration of hospitalization [6]. Laparoscopic procedures have smaller surgical wounds than laparotomies and are considered effective for diagnosis of small bowel obstruction and for finding the cause of obstruction. However, laparoscopic procedure is not suitable in some cases because the relationship of the overall position of the bowel should be confirmed, such as in small bowel volvulus. Hence, this procedure should be performed with caution. Specifically, with post-laparotomy secondary small bowel volvulus, the surgical field may be limited by adhesions and intestinal dilatation within the abdominal cavity. In these cases, surgeons must not hesitate to transition to laparotomy to accurately ascertain the alignment of the intestine.

During laparoscopic procedures, it is possible to examine the treatment guidelines by referencing the aforementioned classifications. Type A often requires intestinal resection due to organic anomalies in the small bowel. Therefore, please consider transitioning to laparotomy, and if possible, the cause should be identified using single incisional laparoscopic surgery and bowel resection with a mini-laparotomy should be performed. For type B cases, there is normally one fixation point. Therefore, if it is possible to secure the surgical field, the adhesion causing the torsion can be separated or a strangulated hernia, which can be reduced laparoscopically. With type C cases, as in our case, we could promptly perform laparoscopic examination. Given that the abdominal surgery procedure history was only a normal free jejunum flap harvest, there was almost no reduction of the operation field due to adhesion to the abdominal wall; hence, the procedure could be completed laparoscopically. However, laparoscopically separating adhesions between different parts of the intestine is an extremely difficult procedure; thus, the procedure must be implemented with extreme care, as it can be associated with intestinal dilatation. Therefore, this procedure should be performed with great care, while always considering the possibility of transitioning to laparotomy. There are few cases of laparoscopic surgery for small bowel volvulus, and more cases are needed to examine indications for this procedure.

## Conclusion

We presented a unique case of secondary small bowel volvulus after harvesting of a free jejunal autograft. Laparoscopic surgery may be useful for treating small bowel volvulus; however, the patient’s treatment indications should be judged carefully.

## Abbreviations

CT: Computed tomography
